# An optimized CT-dense agent perfusion and micro-CT imaging protocol for chick embryo developmental stages

**DOI:** 10.1186/s42490-024-00078-w

**Published:** 2024-04-23

**Authors:** Azza Naïja, Onur Mutlu, Talha Khan, Thomas Daniel Seers, Huseyin C. Yalcin

**Affiliations:** 1 Biomedical Research Center, Qatar University, Doha, Qatar; 2 Petroleum Engineering Program, Texas A&M University, Doha, Qatar; 3 Department of Biomedical Sciences, College of Health Sciences, QU Health, Qatar University, Doha, Qatar; 4 Department of Industrial and Mechanical Engineering, Qatar University, Doha, Qatar

**Keywords:** Chick embryo, Micro-CT, Volume rendering, Heart morphology, Cardiogenesis, Perfusion, Microfil

## Abstract

Compared to classical techniques of morphological analysis, micro-CT (μ-CT) has become an effective approach allowing rapid screening of morphological changes. In the present work, we aimed to provide an optimized micro-CT dense agent perfusion protocol and μ-CT guidelines for different stages of chick embryo cardiogenesis. Our study was conducted over a period of 10 embryonic days (Hamburger-Hamilton HH36) in chick embryo hearts. During the perfusion of the micro-CT dense agent at different developmental stages (HH19, HH24, HH27, HH29, HH31, HH34, HH35, and HH36), we demonstrated that durations and volumes of the injected contrast agent gradually increased with the heart developmental stages contrary to the flow rate that was unchanged during the whole experiment. Analysis of the CT imaging confirmed the efficiency of the optimized parameters of the heart perfusion.

## Background

As the incipient-formed organ, the heart is crucial for healthy embryonic development. The cardiovascular architecture of vertebrate embryos is important for normal function, as the morphology of each chamber contributes to its functional capacity [1]. Cardiogenesis involves the main steps of neural crest migrations, tube formation, looping, trabeculation, and valve development [2]. The use of vertebrate embryo organisms to study the function and development of the human heart has become common practice. These models facilitate the exploration of postulated mechanisms, which may lead to congenital heart diseases (CHDs).

Specifically, avian embryos have numerous advantages within such studies, justifying their use as a developmental model. Firstly, they are more cost-effective and generally more ethically acceptable for use in ex vivo research when compared to other vertebrate organisms (i.e. mammals) [3]. Secondly, the avian cardiogenesis is longer than other available species (i.e. fishes, frogs, and mice), which enables more detailed spatiotemporal analysis and tolerance to microsurgical treatments [4]. Furthermore, it is relatively easy to access the chick embryo since the planar orientation of the embryo on top of the yolk facilitates ready access for imaging and surgical manipulation. Another argument that reinforces the use of chick embryos is that their genome has now been sequenced, showing ~ 70% of homology with the human genome [5]. This high degree of genomic similarity led to the conservation of many key mechanisms in metabolism and development [6] and thus provides the possibility for comparing molecular data with humans [7]. Finally, the avian model is translatable towards cardiac research since both species have four chamber/four valve hearts.

Within biology, the most common morphological analysis is that conducted using transmitted light microscopy of histological sections generated with a microtome. In comparison with these classical sections, newer approaches, such as ultrasound biomicroscopy, micro-MRI, and micro-computed tomography (micro-CT) provide improved resolution and image quality [8, 9]. Micro-CT is now considered as an effective approach well suited towards rapid screening of morphological changes [10–15]. Micro-CT scanners collect a sequence of 2D radiographs (projections) around a radial or helical axis which are reconstructed (typically using filtered back projection or iterative reconstruction) to form a high-resolution 3D (volume) image, with voxel resolutions typically within the tens of micron to sub-micrometer range. These images can provide detailed information on the spatial position and orientation of individual organs, assuming sufficient contrast between the target image features and surrounding materials can be obtained [16]. Current in vivo micro-CT systems are typically few micro-meter resolutions [17, 18] while generalized scanners can reach sub-micrometer resolution [8, 10, 19].

Micro-CT has proved to be effective in the in vivo / ex vivo visualization of the anatomy of small tissues [20–31]). Micro-CT has also been adapted to monitor cardiopulmonary and vascular structures [32, 33], as well as to investigate the toxicity of nanoparticles for preclinical studies using mice [34, 35].

*Butcher and collaborators* were the first to adapt micro-CT imaging towards avian models in order to generate quantitative embryonic cardiovascular volumes at different embryonic stages [10]. Here, a micro-CT dense polymerizing agent was perfused to cardiovasculature for enhanced contrast. Since this groundbreaking study, micro-CT has been used extensively to study cardiovascular morphology in developing chick embryos [4, 9, 36–38]. Specifically, the technique has proved useful for the extraction of accurate volumetric measurements for the determination of embryonic and fetal myocardium properties, particularly following surgical interferences mimicking CHDs [10]. This approach requires the perfusion of a curable polymer-based contrast agent into the subject’s vasculature, with micro-CT scanning and 3D reconstruction for morphological assessment conducted post-polymerization. Avian heart volumes change dramatically during development, meaning that varying quantities of contrast agent need to be injected for developmental different stages.

Additionally, it is crucial to execute the process with precision to prevent vascular rupture caused by heightened pressures during injection. Perfusion should be promptly conducted before the agent commences polymerization.

Inspired by the aforementioned challenges, the objective of this study is to establish an optimized protocol for micro-CT dense agent perfusion and provide micro-CT imaging guidelines tailored to various stages of chick embryo cardiogenesis. Although, several research groups have begun to adopt this approach, the development of an optimized protocol would facilitate its practical adaptation by others, thereby serving as the primary motivation for this research paper.

## Ethical statement

Working with animals requires approval from the ethical committee. Our present work was granted approval from Qatar University Institutional Animal Care & Use Committee (QU-IACUC) under the following approval number: QU-IACUC 022/2020.

## Materials and methods

### Material preparation

#### Microfil cast creation

The microfil cast (Flow-Tech, Carver, MA) was prepared according to the following ratios based on our previous experience: 80% diluent, 15% microfil dye, and 5% curing agent [10]. With this ratio, it requires approximately 30 min for the agent to polymerize, which is sufficient for performing the perfusion procedure on one embryo. For each heart perfusion experiment, we prepared a total volume of 500 μL of microfil cast using the following input volumes of its base ingredients: 400 μL diluent, 75 μL microfil dye, and 25 μL curing agent.

#### Glass capillary preparation

**Fig. 1:**
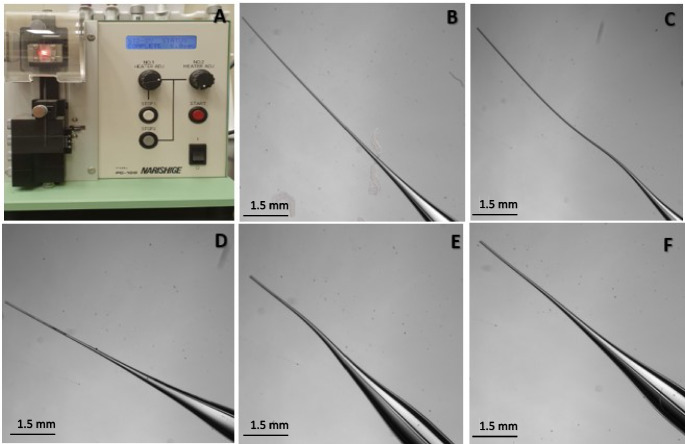
Needles preparation for each embryonic day stage. **A** Puller. **B** needle used at ED3 (HH19) and ED4 (HH24). **C** needle used at ED5 (HH27). **D** needle used at ED6 (HH29) and ED7 (HH31). **E** needle used at ED8 (HH34) and ED9 (HH35). **F** needle used at ED10 (HH36)

To perfuse the heart, specific glass capillaries were prepared for each developmental stage, with different geometries relatable to the puller temperature setting, as shown in Fig. 1. For this, we used a versatile and easy-to-use puller with automated double pulling (NARISHIGE PC-100). For each embryonic stage, we used the needles from the top of the puller (Fig. 1).

Changes in puller temperature result in needles with different sizes: as the puller temperature increases, the glass capillary tip size increases commensurately. Table 1 presents the different puller temperatures matched with their corresponding embryonic stage and tip sizes.

**Table 1:** Heater temperatures and tip sizes for the different chick embryo stages

	**Temperatures**	**Tip sizes (microns)**
**ED3 (HH19)**	71	41.41
**ED4 (HH24)**		
**ED5 (HH27)**	80	49.64
**ED6 (HH29)**	82	51.07
**ED7 (HH31)**		
**ED8 (HH34)**	83	53.09
**ED9 (HH35)**		
**ED10 (HH36)**	85	59.8

Other key specifications for the needle micromanipulator are the adjustment of its plates: Plate 1 and Plate 2, which are fixed at 3.5 and 7 mm respectively. In addition, the puller is loaded with three heater blocks: two heavy blocks and one light block. While these settings are for this specific puller, a similar approach can be used for other pullers to obtain similar capillaries. The dimensions of the capillary tubing used in the needle fabrication procedure are as follows: 4 mm thin wall; 1.5 mm OD/1.12 mm ID.

## Methods

### Experimental design

In the present work, we have analyzed a total number of 240 chick embryos from ED3 (HH19) to ED10 (HH36). For each ED, we included 30 embryos for each measurement. Fertilized eggs are delivered from a local poultry (Arab-Qatari Poultry Farm, Doha, Qatar). Ideally, eggs should be used fresh and directly incubated at 37 °C with a fully filled water tank to provide 60% of humidity. As a precautionary measure against any unforeseen requirement to postpone the experiments, eggs are kept in a 13 °C cooler for a maximum of 7 days. Inside the incubator, the eggs are rocked for 3 days.

Details of our culture protocols can be found in our previous work, but are explained briefly here [36, 39]. Embryonic chicks develop on top of the yolk sac for the first five days before sinking into the middle of the egg. On day three post-incubation (ED3), eggs are taken out of the incubator and transferred to another rack in which they are kept horizontally for at least 2 min to allow the embryo to migrate to the top of the egg. Once relocated, a small hole was made at the pointed end of the egg after wiping the shell with ethanol (70%). Using an angle of ~ 45°, a syringe needle is placed inside the egg and aspirated between 5 and 6 mL of the albumin. Another hole is made near the drawn circle then, using a curved scissor, a window is made following the demarcated circle on the eggshell. It is recommended to use the scissors carefully to avoid any contact between the scissor edges and the yolk. Unfertilized eggs, stinking embryos to the shell, and undeveloped embryos are discarded while the hole of the fertilized and intact egg is sealed with transparent tape to prevent dehydration. Embryos are then re-incubated in the bench-top incubator under the same incubation conditions previously set (temperature: 37.5 °C and humidity: 60%). Eggs are cultured for up to seven additional days (end of the experiment at day 10) to allow further development to targeted developmental stage.

**Fig. 2:**
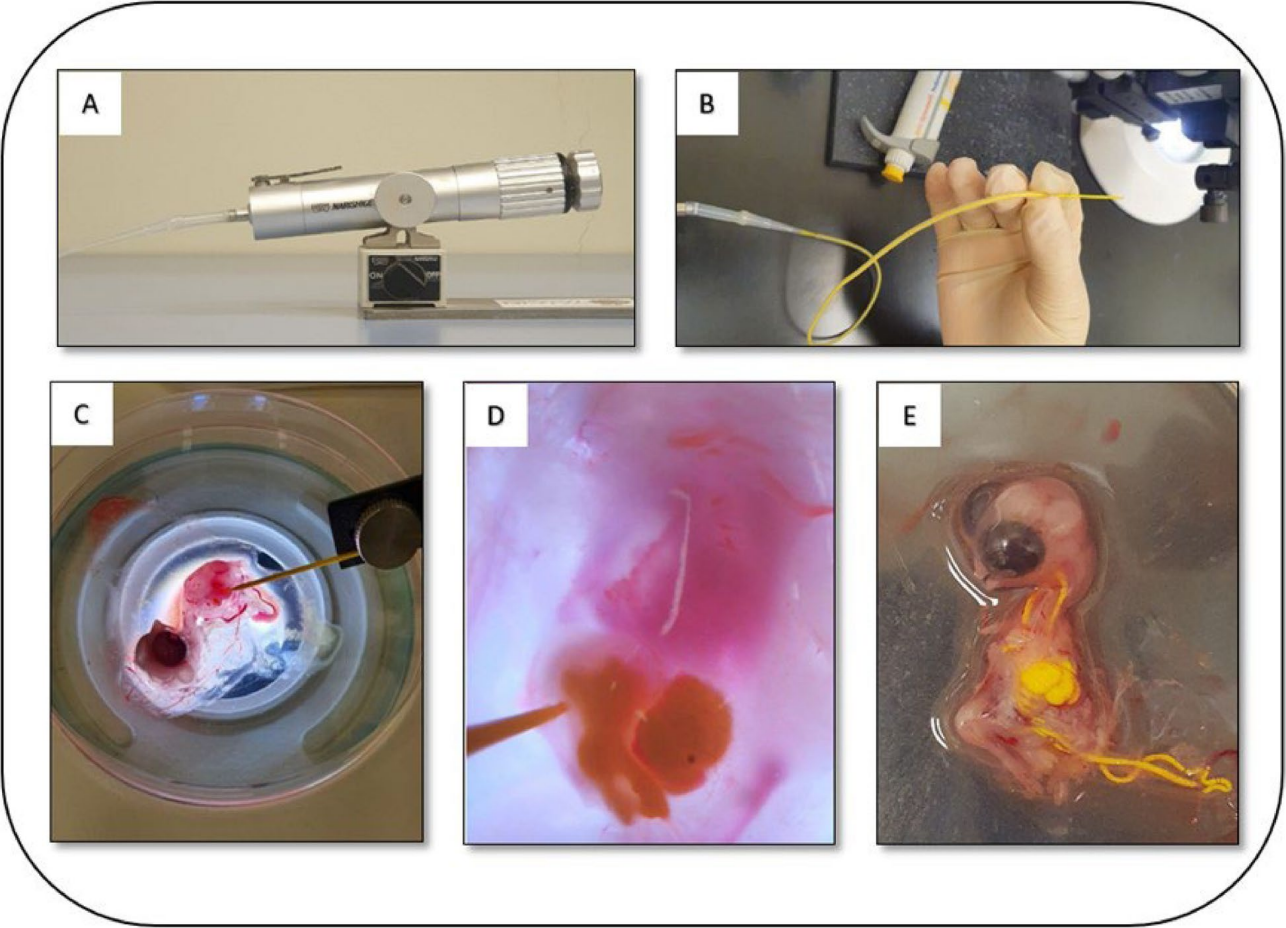
Perfusion steps. **A** The manual pump used for microfil dye perfusion. **B** The glass capillary is drawn into the microneedle and filled with the microfil dye. **C** The glass capillary is positioned toward the heart. **D** The heart during the microfil dye perfusion experiment. **E** The heart filled with the microfil dye

The perfusion experiment was conducted over a total period of 7 days where the embryos were at the following embryonic days: ED3, ED4, ED5, ED6, ED7, ED8, ED9, and ED10. Prior to separation from eggs, embryos were not subject to any euthanization protocol. Beating of the heart is desired during perfusion so that contrast agent is pumped to the outflow tract with heartbeats as long as possible. At each embryonic day, the experiment started by separating the embryo from the egg via cutting the yolk blood vessels surrounding the embryo. Vessels should be cut from as far from the embryo as possible to provide some volume reservoir during the injection. The embryo is then transferred to a petri dish and placed under a stereomicroscope connected to a camera (Zeiss, Axiocam ERc 5s). Blood vessels should be kept intact during this transfer. Using a micro-pump (NARISHIGE) (Fig. 2A), the glass capillary is drawn into the microneedle (WPI; ID: 1/32", OD: 3/32") filled with the microfil dye, prepared using the specifications described above (Fig. 2B). The needle is oriented toward the heart and inserted gently into one of the ventricles after crossing the pericardium (Fig. 2C). Once inside, dye is injected carefully into the heart using a low flow rate ranging between 2.8 and 6.8 µL/min, depending on the embryonic stage (Fig. 2D). To optimize the chances of a successful perfusion experiment, we kept the heart beating as much as possible using a few drops of warm PBS to allow the dye to cover all heart compartments. Once the heart is completely filled with the microfil dye (Fig. 2E), the embryo is kept in a petri dish for at least 30 min at room temperature for perfusion agent polymerization before being transferred to tubes containing PFA (4%).

### Duration measurement

The duration of the perfusion procedure is monitored using a timer. The experiment initiates with the insertion of the glass capillary tip into the heart and the injection of the first drop of dye therein and terminates when the heart, major blood vessels within the embryo and the vitelline vessels are fully perfused with the dye. At this point, the micro-pump pressure is released and the capillary needle is pulled back. The dye should fill both intraembryonic and extraembryonic vessels to provide constant pressure to the heart so that the heart is kept fully perfused. However, over-perfusing the embryo should be avoided, since this may result in the blood vessels bursting and leakage of the dye. As a guide, once the blood vessels proximal to the eye are perfused, the process can be slowed down or stopped.

### Volume measurement

To calculate the volume of the perfused heart, we mark the initial volume of the dye that appears inside the micro-tubing using a waterproof marker. At the end of the perfusion, we pull back the needle from the heart and stop the perfusion. We mark the level of the dye in the micro-tubing, and then calculate the length of the micro-tubing used to perfuse the whole heart.

The volume calculation is as follows:

$${\text{Volume}}=((\uppi * {{\text{D}}}^{2}) / 4)\ *\mathrm{\ length\ }(\mathrm{\mu L})$$

D: the inner diameter of micro-tube.

Length: length of micro-tube used to fill the heart.

#### Flow rate measurement

The flow rate of the heart perfusion process is calculated as follows:

$$\mathrm{Flow\ rate }=\mathrm{ volume}/\mathrm{time\ }(\mathrm{\mu L}/{\text{min}})$$

### Micro-CT imaging protocol

Once the perfusion agent polymerizes after ~ 30 min, embryos are fixed in 4% paraformaldehyde for 24 h and then, transferred to PBS-filled tubes. ED3 (HH19) to ED5 (HH27) embryos are placed in 3 ml cryotubes (Nalgene), whereas ED6 (HH29) to ED10 (HH36) embryos are placed in 15 ml centrifuge tubes. In order to stabilize the embryos during scans, a small piece of foam is placed inside each tube.

Embryos are loaded into x-ray transparent capillaries and imaged using the Thermo-Fisher Mk1 Heliscan x-ray microcomputed tomography scanner located at Texas A&M University at Qatar using a space-filling helical scan protocol and beam energy ranging between 80–90 keV. Hope you are well. Stainless steel filter of thickness 0.1mm was used at the source and an Aluminum filter with a thickness of 2mm was used at the detector to minimize beam-hardening artifacts, with the reconstructed image voxel resolution ranging between 7.6–14 µm (governed by the diameter of the sample chamber used). Finally, reconstructed voxel images are denoised using the edge-preserving non-local means (NLM) filter.

### Processing micro-CT images for morphological measurements

**Fig. 3:**
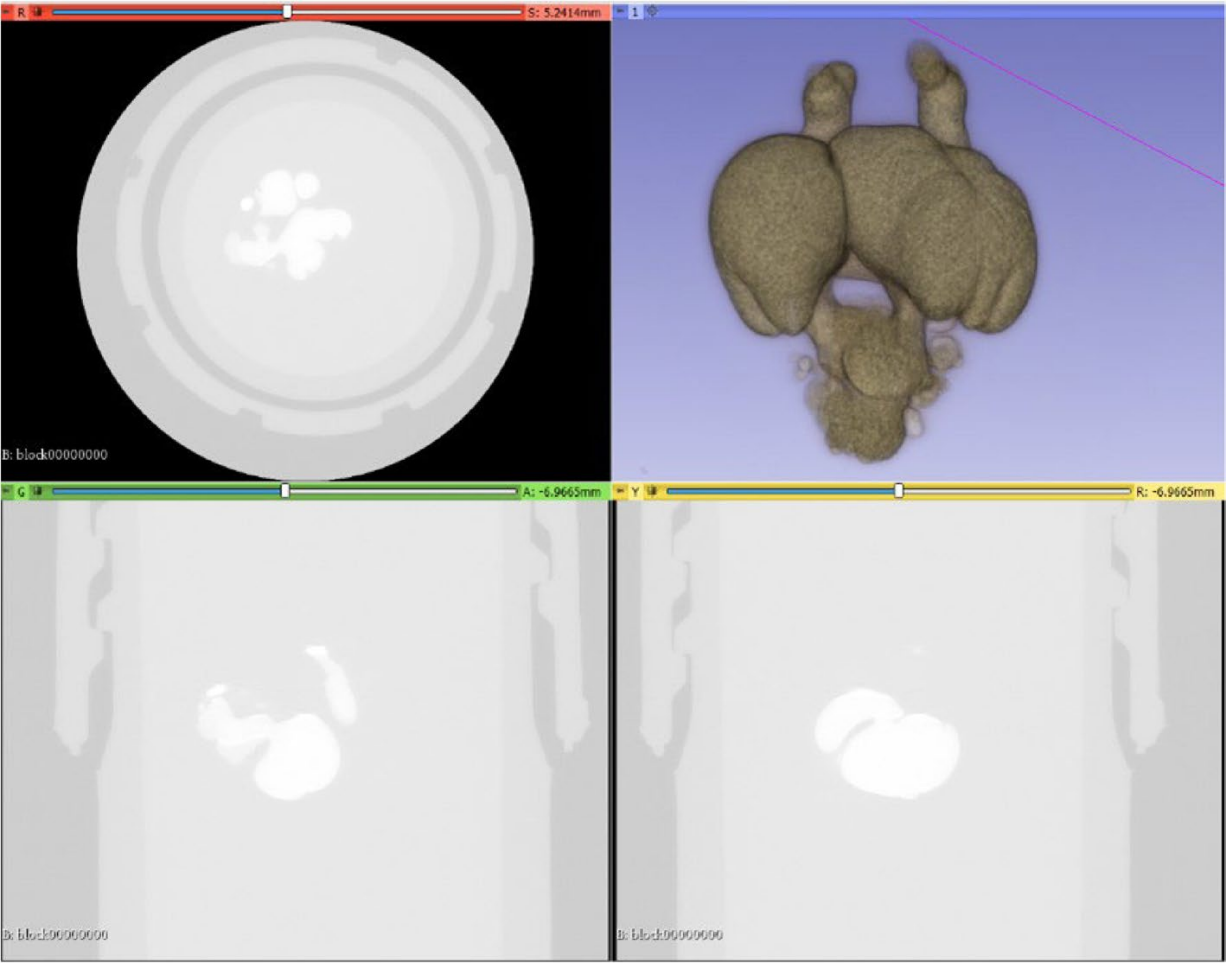
Importing raw micro-CT images into 3D Slicer

Using micro-CT, features that cannot be segmented or interrogated with standard medical CT machines can be characterized in detail due to the high spatial resolution of such systems. In this work, perfused chick embryo micro-CT images were generated in three axes with 7 to 14-µm layer intervals, dependent upon the volume and stage of the embryo under investigation. The ED 3, 4, 5, 6, 7, 8 and 10 were segmented using open-source 3D Slicer software (www.slicer.org). NLM-filtered grayscale micro-CT images are imported into 3D Slicer software (Fig. 3) for segmentation and visualized in 3D using volume rendering.

**Fig. 4:**
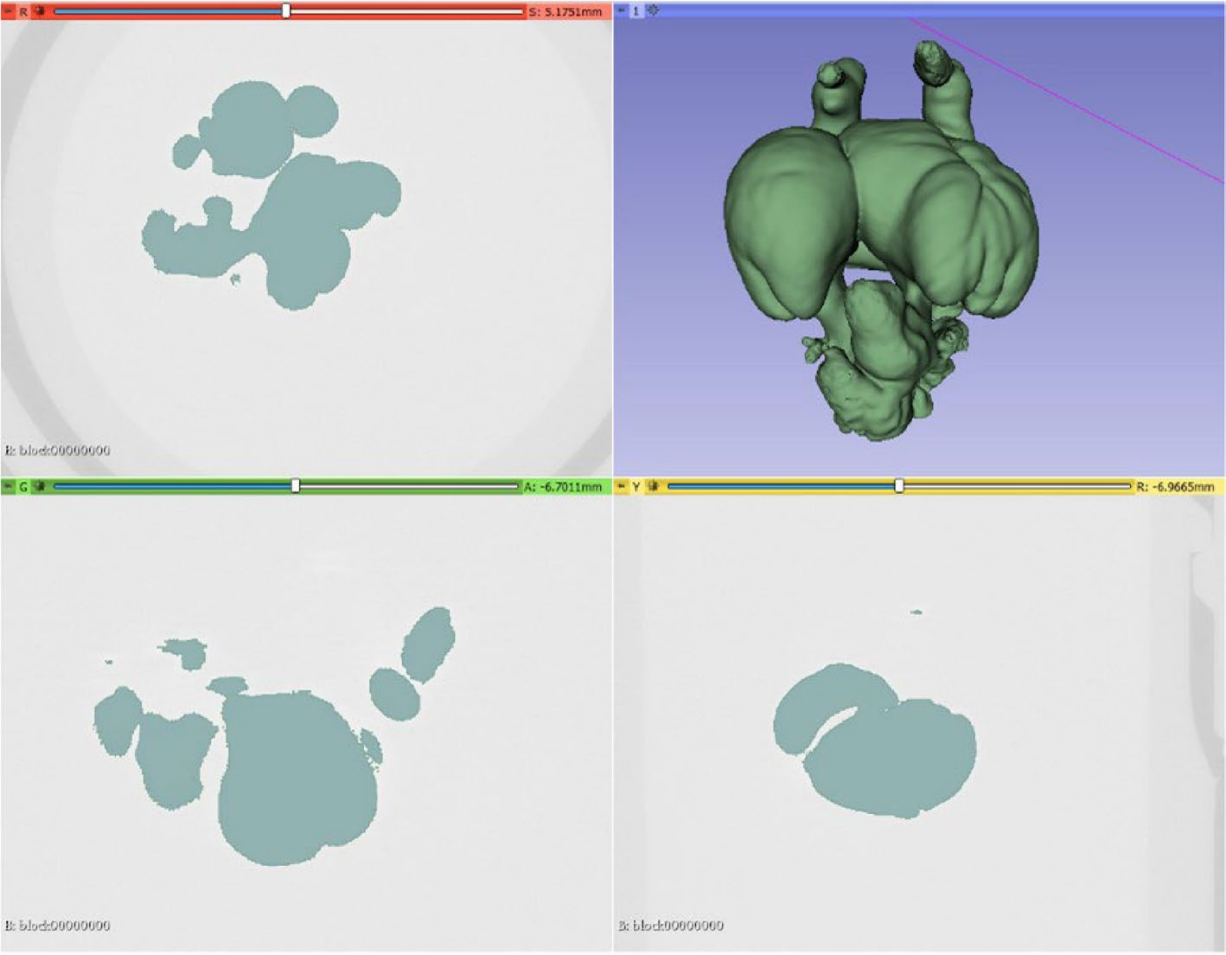
Segmented 3D volume with obtained threshold value

**Fig. 5:**
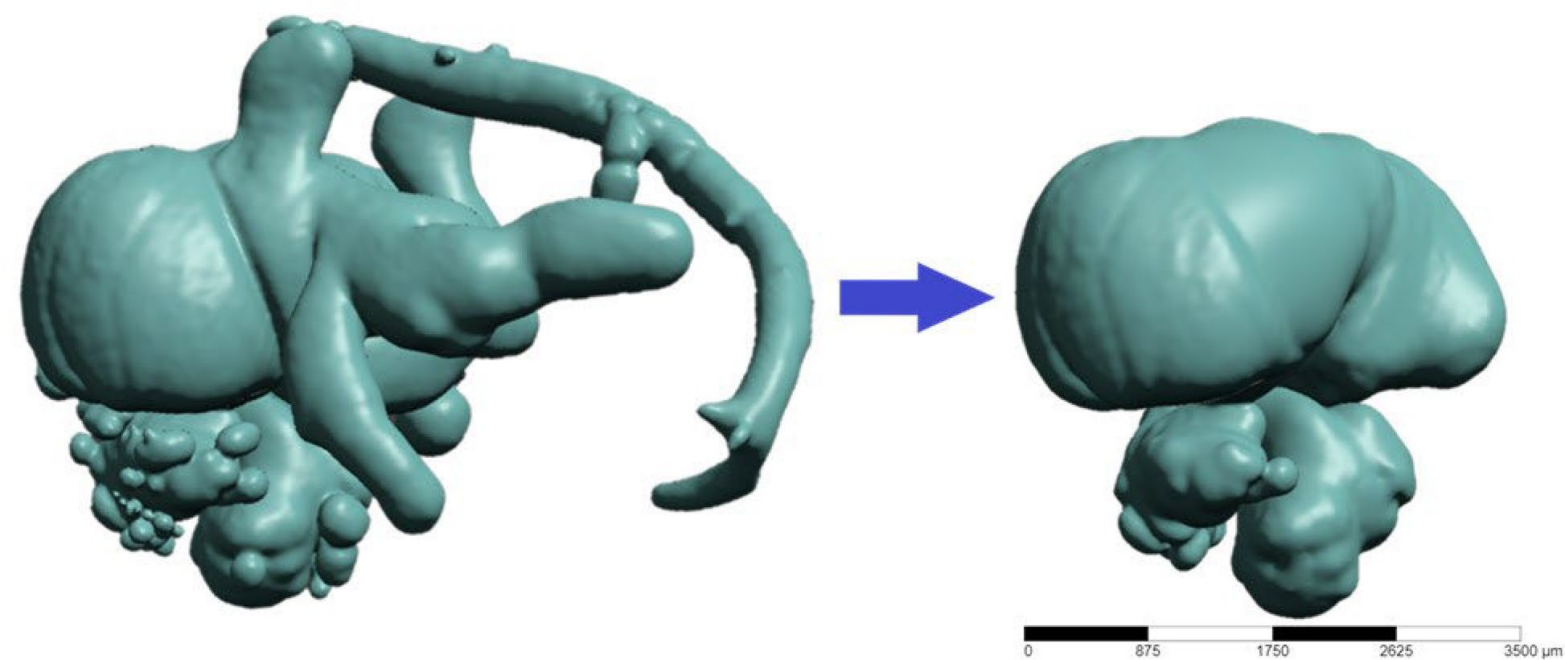
An example unedited and cleaned chick embryo hearth geometry (ED5)

Binary segmentation of the micro-CT image volumes is required for further analysis and modeling. In this work, we utilize interactive thresholding to generate binary image sets from grayscale micro CT images, whereby a threshold value is manually selected and applied to separate the target image features (i.e. micro-dye perfused cardiovascular elements) from background materials (Fig. 4). Post-segmentation, there may be extraneous features, artifacts, or under-segmented regions within the binary image. Consequently, geometries segmented in “.stl” format were subjected to cleaning using Mesh Mixer (http://www.meshmixer.com/Autodesk, Inc., San Rafael, California, USA), which was used to remove the noise in the segmented geometry and to retain only the atria and ventricle parts of the heart (Fig. 5).

## Results

### Duration, volume and flow rate of perfusion for different stages

Effective perfusion is critical within this protocol. The duration of the procedure should be limited, since the polymer may begin to cure during perfusion if performed too slowly (i.e. over the course of tens of minutes). However, if the perfusion is performed overly aggressively at high flow rates, the heart or blood vessels may burst due to excessive pressure, jeopardizing the experiment. The heart should be fully perfused with extra dye in the blood vessels so that a constant pressure will be supplied to the heart even after polymerization commences, in order to keep the heart fully dilated. Given the above, the most important parameters for the perfusion protocol are the duration of the perfusion, the total volume to be perfused, and the flow rate of the perfusion.

**Fig. 6:**
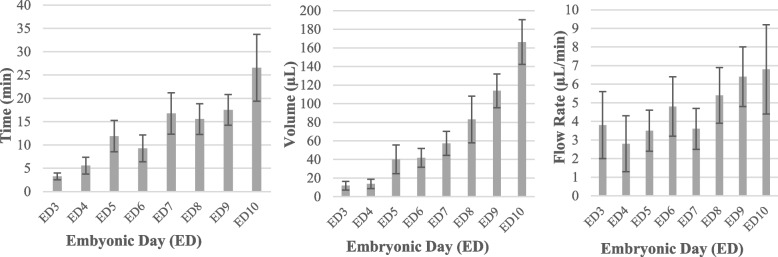
Duration, volume, and flow rate of the perfusion in chick embryo hearts at different stages. **A** Duration of the perfusion. **B** Volume of the perfused dye. **C** Flow rate of the perfusion. Each bar represents mean ± SD of 30 perfused hearts

Figure 6 shows the profiles of the duration of perfusion, total volume to be perfused, and flow rate of perfusion for different embryonic stages. Table 2 summarizes the average values of each target parameter for each developmental stage. Over the whole experiment, the duration and volume of the perfusion gradually increase with the offstage of chick embryos. Though ED3 (HH19) required approximately 4 min to be saturated with the microfil dye, ED10 (HH36) perfusion needed more than 26 min (Fig. 6A, Table 2), due to the dramatic increase in heart volume achieved between these two developmental stages. The required perfusion durations and volumes of the perfused microfil dye gradually increased with the developmental stage (Fig. 6B). Starting with an average of 11.8 µL at ED3 (HH19), the perfused microfil dye reached its maximum at ED 10 (HH36), with an average volume of 166.3 µL (Table 2). Flow rate of perfusion does not significantly over the studied developmental stages due to hard limits placed upon flow rate, required to promote the integrity of each subject’s cardiovascular system. Specifically, flow rates ranged between 3.8 to 6.8 µL min^−1^ (Table 2).

**Table 2:** Mean values of duration (min), volume (µL), and flow rate (µL. min^−1^) of the perfusion process. Data is represented as Mean ± SD of 30 perfused hearts for each developmental stage

	**Duration ± SD**	**Volume ± SD**	**Flow rate ± SD**
**ED3 (HH19)**	3.24 ± 0.72	11.8 ± 0.47	3.8 ± 0.18
**ED4 (HH24)**	5.57 ± 1.79	13.7 ± 0.51	2.8 ± 0.15
**ED5 (HH27)**	11.89 ± 3.38	40.1 ± 1.54	3.5 ± 0.11
**ED6 (HH29)**	9.26 ± 2.86	41.8 ± 1.01	4.8 ± 0.16
**ED7 (HH31)**	16.76 ± 4.45	57.3 ± 1.29	3.6 ± 0.11
**ED8 (HH34)**	15.56 ± 3.29	83 ± 2.51	5.4 ± 0.15
**ED9 (HH35)**	17.51 ± 3.29	113.8 ± 1.81	6.4 ± 0.16
**ED10 (HH36)**	26.55 ± 7.17	166.3 ± 2.4	6.8 ± 0.24

### Generated micro-CT 3D geometries at different developmental stages

**Fig. 7:**
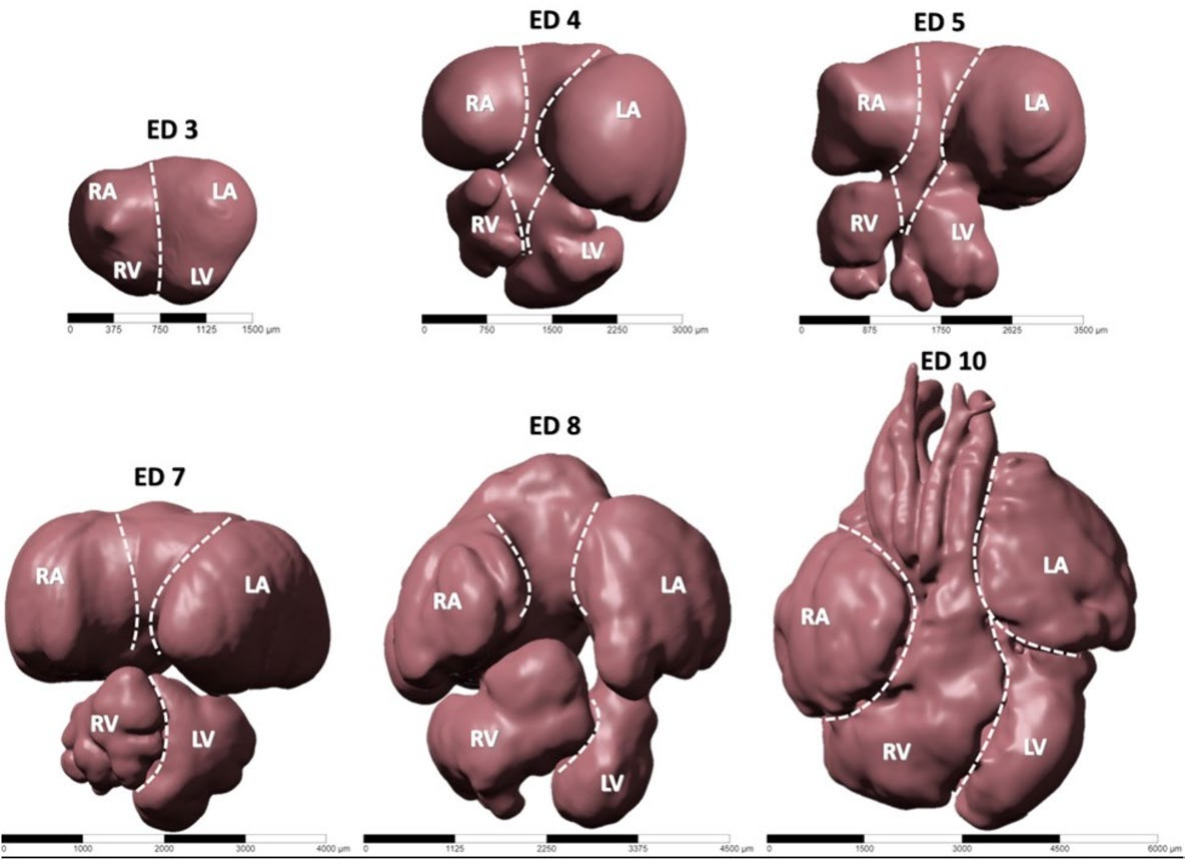
High-resolution micro-CT scans of chick embryonic hearts at different developmental stages. Crossed lines demarcate chamber borders. RA: right atria, LA: left atria, RV: right ventricle, LV: left ventricle

Scans of perfused hearts at different stages can be seen in Fig. 7. Anatomical features can be easily extracted from these high-resolution 3-D reconstructed images. Whilst ED3 (HH19) exhibits an unseptated heart, septation initiates at ED5 (HH27) and continues over later stages. At ED10 (HH36), morphological development is almost complete.

## Limitation of the procedure

The optimized protocol involves several steps aimed to perfuse the heart of chick embryos with CT dense agent at different developmental stages. The protocol’s focus on embryos from ED 3 to ED10 which may not capture the full spectrum of developmental changes occurring in chick embryos. Additional time points could provide a more comprehensive understanding of embryonic development but may require adjustments to the experimental procedures.

Potential threats, difficulties, and considerations in the protocol include challenges in egg handling and incubation, as keeping eggs in a cooler for up to 7 days before incubation may threaten embryo viability or development, and maintaining consistent humidity and temperature levels in the incubator is crucial. During the perfusion experiment, cutting the yolk blood vessels and transferring embryos to petri dishes can cause damage or stress, while maintaining embryo heartbeats may be challenging, potentially leading to bursting blood vessels through over-perfusion. As previously mentioned, the ratio utilized in this study to prepare the microfil cast necessitates approximately 30 min for the dye to polymerize. At ED10, certain perfusions exceeded the 30-min mark for complete heart perfusion, posing a risk of premature polymerization of the microfil dye agent. In micro-CT imaging, improper fixation or handling of embryos before imaging may introduce artifacts or distortions, and achieving consistent imaging parameters across different embryos and stages is difficult. Processing micro-CT images for morphological measurements poses challenges as inaccurate segmentation or cleaning may lead to errors, and manual thresholding and cleaning are subjective and time-consuming, making consistency across samples difficult. Despite efforts to minimize artifacts during micro-CT imaging, factors such as fixation quality, sample orientation, and beam hardening can contribute to image distortions and inaccuracies, compromising measurement accuracy. Additionally, manual thresholding and cleaning of micro-CT images introduce the potential for bias and inconsistency in segmentation and analysis, affecting measurement reliability.

## Possible and acceptable deviations

While deviations should be minimized to ensure result consistency, there are circumstances where adjustments may be necessary or acceptable. These include modification of the perfusion technique, where variations in needle size or injection rate may be acceptable depending on the developmental stage of the embryos as described above. Optimization of image processing through alternative techniques or software tools may also be employed to improve efficiency and accuracy, provided they are validated to ensure reliability.

## Conclusion

In this study, we have presented an optimized micro-CT dense agent perfusion protocol using chick embryos as biological models. Perfusion duration and volume of the injected contrast agent gradually increased with the heart developmental stages. Once fixing sizes of the glass capillaries used to perfuse the hearts of chick embryos, the flow rate of the perfusion seemed to be unchanged within all the tested stages which means that fixing the parameters of the perfusion are very important in the experiment. The CT imaging guidelines showing 3D heart models and providing high resolution and visualization of the heart confirm the efficiency of the optimized parameters of the perfusion. The procedure is challenging when applied to chick embryos prior to ED3, because of the small size of the targeted heart. At later stages, successful perfusion can be easily achieved once the procedure is applied carefully. This protocol can be applied to different experimental models such as Zebrafish, by optimizing the perfusion parameters according to the lumen volume of the cardiovascular system.

## Data Availability

Materials described in the manuscript, including all relevant raw data, will be freely available to any scientist wishing to use them for non-commercial purposes.
